# Applications of Artificial Intelligence in Transcatheter Aortic Valve Replacement: A Review of the Literature

**DOI:** 10.3390/life15111724

**Published:** 2025-11-07

**Authors:** Flora Tsakirian, Dimitrios Afendoulis, Andreas Mavroudis, Svetlana Aghayan, Maria Drakopoulou, Andreas Synetos, Sotirios Tsalamandris, Konstantinos Tsioufis, Panayotis Vlachakis, Konstantinos Toutouzas

**Affiliations:** Unit of Structural and Valvular Diseases Heart Diseases, First Cardiology Department of Cardiology National Kapodestrian, University of Athens, General Hospital of Athens “Ippokratio”, 11527 Athens, Greece; loratsakirianmed@gmail.com (F.T.); andreasmavroudis89@gmail.com (A.M.); dr.svetlana.aghayan@gmail.com (S.A.); mdrakopoulou@hotmail.com (M.D.); synetos@yahoo.com (A.S.); stsalamandris@hotmail.com (S.T.); ktsioufis@gmail.com (K.T.); vlachakispanag@gmail.com (P.V.); ktoutouz@gmail.com (K.T.)

**Keywords:** TAVI procedure, artificial intelligence, machine learning tools

## Abstract

Introduction: Artificial intelligence (AI) tools have emerged in cardiovascular clinical practice. Regarding transcatheter aortic valve replacement/implantation (TAVR/TAVI) procedures, their utilization optimizes procedural planning, aids physicians with decision making, and predicts possible post-procedural complications. Moreover, machine-learning (ML) models, compared with traditional mortality risk scores, show promising results considering predicted mortality in TAVI patients. However, further validation is required. As the implementation of cardiovascular procedures can be challenging, AI technology broadens the armamentarium of tools that a clinician is able to use for a more comprehensive evaluation of patients, minimizing complications and resulting in optimum clinical outcomes. Methods: A comprehensive literature search was conducted through the PubMed and Google Scholar databases from inception to 20 September 2025, to identify relevant studies. The search strategy included the following keywords: [“TAVI” OR “TAVR”] AND [“AI”, Artificial Intelligence]. Results: According to our database research, 7177 articles were initially screened, and 2145 duplicate articles were excluded. Eventually, 189 articles were evaluated by our reviewers and 51 articles of studies published between 2014 and 2025 were included in our review. Conclusions: AI algorithms could revolutionize the Heart Team decision making process, being not only a tool for patient evaluation but an active member of the team with applications to analyze and optimize all stages of the TAVI procedure, guide decision making and predict outcomes, and, with the contribution and evaluation of information from all human members of the team, enhance even more the patient-mediated medicine/interventions.

## 1. Introduction

Artificial intelligence (AI) tools are increasingly used in cardiovascular clinical practice. Specifically for the evaluation of transcatheter aortic valve replacement/implantation (TAVR/TAVI) candidates, the AI-assisted estimation of anatomical markers in computed tomography (CT) analysis (e.g., aortic annulus measurement and orientation, coronary ostia height, coronary artery calcifications, calcium quantification and distribution) approximates clinician judgment, contributing to better accuracy considering valve size prediction even in complex, heavily calcified anatomies. Deep-learning reconstruction of CT images can also contribute to substantial reduction of pre-TAVI CT radiation while preserving diagnostic accuracy. Τhe evaluation of pre-TAVR procedure CT images can offer further insights beyond anatomy, contributing to the prediction of probable complications after the TAVR procedure and, additionally, having a prognostic role. Nowadays, the integration of artificial intelligence into interventional cardiology has demonstrated substantial progress. The proposed AI framework described by recent IEEE research achieved up to 25–30% improvement in predictive accuracy compared with traditional models. The combination of multi-modal imaging and clinical data also reduced procedural planning time by nearly 40%, achieving greater workflow efficiency and precision. Moreover, the study emphasized the need for real-time implementation and interpretability to ensure safe, transparent, and clinically reliable integration of AI tools into cardiovascular practice [1].

## 2. Methods

### 2.1. Research Strategy

The current review was reported in accordance with the preferred reporting items for systematic reviews (PRISMA). A comprehensive literature search was conducted through the PubMed and Google Scholar databases from inception to 20 September, 2025, to identify relevant studies. The search strategy included the following keywords: ‘Transcatheter Aortic Valve Implantation’, ‘TAVR’, ‘Aortic Valve Stenosis’, ‘TAVI’, ‘Artificial Intelligence’, ‘machine learning’, ‘neural networks’. Boolean research terms included [“TAVI”/“TAVR”] AND [“AI”, Artificial Intelligence], [“TAVI”/“TAVR”] AND [‘Machine Learning’], [“Aortic Valve Stenosis”] AND [“AI”, Artificial Intelligence], [“Aortic Valve Stenosis”] AND [‘Machine Learning’]. Synonyms and equivalent terms for these keywords were also included, as well as reference lists of the articles included in the review were screened for additional citations, to ensure broad research.

### 2.2. Eligibility Criteria, Screening, and Data Extraction

Studies were eligible for inclusion if they focused on TAVR, TAVI, or Aortic Stenosis and the use of AI algorithms. This included the application of machine-learning algorithms, neural networks, deep learning, hybrid models, or AI reconstruction algorithms for predicting post-TAVR outcomes, mortality and complications, planning and guiding of the procedure, decision making assisting tools and applications on imaging modalities, and image quality enhancement. Exclusion criteria included abstracts, editorials, case reports, and animal studies, or non- English articles. Moreover, any articles not relevant with the application of AI algorithms, machine-learning models, or neural networks on TAVI procedural aspects, or aortic stenosis management, were excluded from our review. After removing duplicate articles, all available articles were screened for titles and abstracts relevance. Furthermore, full texts of potentially eligible studies were assessed by the same reviewers. Key data were extracted by the reviewers, focusing on study and population characteristics, AI methodologies and algorithms, imaging modalities, and types of outcomes predicted by the application of machine-learning algorithms for TAVI procedure.

All titles and abstracts were screened independently by two reviewers of our team (T.F, A.D), and data extraction was performed. Any conflicts were reviewed by the inclusion of one separate expert reviewer (S.T) and resolved by consensus of the whole team. Methodological quality was assessed by two more reviewers. Any disagreements regarding classifications were resolved through consensus among the reviewers.

Two separate reviewers (A.S, A.M) evaluated the quality and risk of bias of the included studies using the prediction model risk of bias assessment tool (PROBAST), which assesses studies in four domains: participants, predictors, outcome, and analysis.

## 3. Results and Discussion

**Figure 1:**
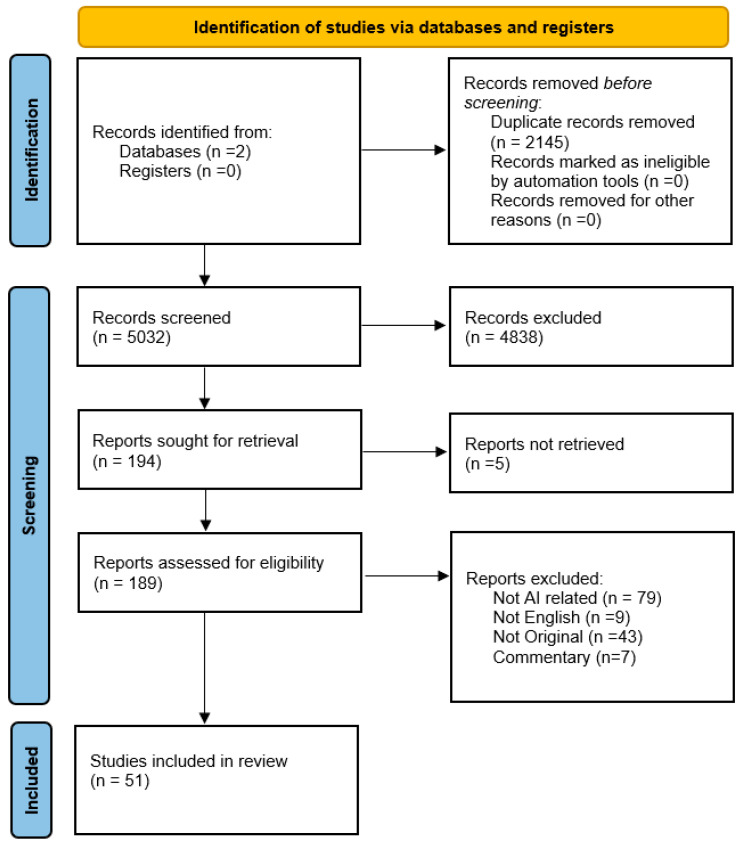
PRISMA Flowchart.

According to our database research, 7177 articles were initially screened, and 2145 duplicate articles were excluded. Eventually, 189 articles were evaluated by our reviewers, and 51 articles of studies published between 2014 and 2025 were included in our review (Table 1). The PRISMA flowchart for our selection process is presented (Figure 1).

### 3.1. AI in Imaging and Pre-Procedural Planning

#### 3.1.1. Automated CT Segmentation, Landmarking, and Measurement

With the use of AI and subsequently deep-learning systems nowadays, we are able to automate core pre-TAVR CT tasks, which may include root/annulus segmentation, landmark localization, measurement of annular perimeter-derived diameter, coronary ostia height, calcification burden, and aortic angulation with almost high-level accuracy. In a multicenter study, an end-to-end pipeline achieved impressive diagnostic precision (accuracy 0.989, sensitivity 0.979, specificity 0.986) while standardizing output and minimizing the time needed for analysis [2]. Additional reviews outline how these models reduce observer variability and support valve sizing and placement simulations, including extended reality (XR)-assisted visualization for complex anatomies and multidisciplinary team (MDT) planning [3,4]. Conceptually related work emphasizes that AI-enabled screening and planning should augment rather than replace clinician judgment, particularly given bias and validation concerns [5,6,7]. A recent study further demonstrated that deep learning can significantly enhance 3D CT reconstructions for TAVI, producing sharper, more reliable images and improving valve size prediction accuracy to nearly 90%, with excellent sensitivity and specificity [8]. Purpose-built segmentation frameworks (e.g., cascaded CNNs) further report sub-millimeter landmarking accuracy and rapid runtimes (seconds to <30 s), enabling reliable annulus orientation and diameter estimation within ~2 mm of expert measurements [9,10,11]. More recent studies confirmed these benefits, showing that AI-driven CT frameworks achieve strong agreement with expert manual measurements (>0.97 accuracy, sensitivity, specificity) [12], while dedicated vessel-assessment tools matched radiologists within ±2 mm across key landmarks in a fraction of the time [13]. In addition, deep-learning CT reconstruction has been shown to improve lumen visualization and reduce artifacts in heavily calcified anatomies, enhancing diagnostic confidence and procedural planning [14]. A recent review also highlighted automated platforms such as TAVI-PREP and 4TAVR, which extract essential measurements within minutes and standardize 3D reconstructions, though broader validation across diverse anatomies is still required [15].

#### 3.1.2. Quantifying Calcification and Anatomic Complexity

Machine-driven measurement of calcium scoring, including volume and distribution of calcium, is increasingly reliable, aligning well with expert reads while improving speed and reproducibility, enabling proper sizing and complications risk stratification for paravalvular leak or annular injury [16,17,18]. “Digital twin” pipelines such as CardioVision reconstruct patient-specific root geometry and map calcification without manual delineation, advancing pre-implant modeling and optimizing device deployment planning. Allowing further external validation across diverse anatomies is the next step [19].

#### 3.1.3. Body Composition, Opportunistic Imaging Biomarkers, and CT-Derived Physiology

AI has diversified the applications of CT in body mapping. Automated L3 body-composition profiling (sarcopenia; fat density/quality) has been documented to correlate with survival after TAVI, offering readily available prognostic signals from routine scans [20,21]. An AI-derived Left Atrioventricular Coupling Index (LACI)—ratio of left atrium (LA): left ventricle (LV) end-diastolic volumes from fully automated analysis—was independently associated with mortality (cut-off ~43.7%), even after STS-PROM adjustment and in preserved ejection fraction (EF) subgroups, implying added pathophysiologic insight [22]. Opportunistic vertebral volumetric bone mineral density (vBMD) from TAVR CT flagged osteoporosis (~43% prevalence), showed high diagnostic accuracy (AUC ~0.96 for thoracic vBMD), and identified patients with expected survival status of less than 1 year. This pattern may represent a frailty-related risk profile, highlighting patients who are more vulnerable to adverse outcomes [23]. Epicardial fat quantified on CT is associated with baseline atrioventricular (AV) conduction abnormalities and higher rates of permanent pacemaker implantation, adding a biologic-risk layer to pre-TAVR assessment [24]. CT-based pressure gradient estimation using AI also shows promising results, hinting at non-invasive physiologic surrogates to complement planning [25].

#### 3.1.4. 3D/4D Analysis, Deformation Tracking, and Post-TAVI Assessment

AI-driven 4D-CT tools quantify dynamic prosthesis behavior—volume, cross-sectional area, and displacement across the cardiac cycle, facilitating standardized post-TAVI surveillance and durability research [26,27]. These dynamic markers may help uncover device–tissue interactions that static imaging misses.

### 3.2. Image Quality Optimization: AI Reconstruction, Coronary Rule-Out, and Protocols

#### 3.2.1. AI-Enabled CT Reconstruction and Dose Reduction

Deep-learning reconstruction (DLR/AI-IR) facilitates radiation-dose reduction while preserving or even improving diagnostic quality. Prospective and cohort studies report lower noise, higher contrast-to-noise (CNR)/single-to-noise (SNR), and maintained measurement fidelity (e.g., annulus sizing) despite low-tube-voltage protocols [18,28,29]. On standard 8 cm detector scanners, combining DLR approaches cut effective dose by ~50%+ with equal or better image quality for valve and access assessment, particularly valuable in elderly, comorbid cohorts [29]. Earlier protocol-optimization work already showed that 70–100 kV scanning yields good-to-excellent quality with large dose savings for both coronary and aorto-iliac computed tomography angiography (CTA), supporting the subsequent gains seen with DLR [30]. In addition, a prospective study of 109 patients demonstrated that AI-driven iterative reconstruction (AIIR) produced sharper images with the highest signal, contrast, and lowest noise compared with both standard- and hybrid-reconstruction methods, all while reducing radiation exposure. This confirms that AI-based reconstruction can not only preserve but also enhance diagnostic quality in pre-TAVI imaging, making evaluations both safer and more precise [31].

#### 3.2.2. Coronary Evaluation During TAVR Work-Up

Photon-counting CT and AI-augmented coronary analysis continue to mature [16,17]. In a TAVR cohort with properly imaged coronaries, a deep-learning tool achieved 100% sensitivity and negative predictive value (NPV) for ≥50% stenosis (specificity ~39%), approaching radiologist sensitivity and supporting the use-case of safely ruling out significant CAD to downgrade invasive angiography when negative [32]. Reviews echo how AI is being embedded across the CCTA pipeline (acquisition, reconstruction, decision support) to accelerate and optimize coronary assessment alongside TAVR planning [33].

### 3.3. Predictive and Decision-Making Models

#### 3.3.1. Mortality Prediction: Single-Study Signals and Pooled Evidence

Across multiple cohorts, ML models outperform legacy scores (EuroSCORE II, TAVI-SCORE, CoreValve). A Gradient Boosting Machine surpassed TAVI-2 and CoreValve for 1-year mortality, illustrating the value of non-linear learning on routine variables [34]. A pilot TAVI study further showed that two simple pre-procedural indices—ASA class and Clinical Frailty Scale—outperformed EuroSCORE II (AUC ~0.80 vs. ~0.66) and also stratified length of stay and costs into clinically meaningful tiers, highlighting their value as inexpensive and accessible predictors [35]. In addition, other studies investigated the value of integrating clinical data with imaging features, showing that machine-learning models provided higher predictive accuracy than conventional risk scores for both short- and long-term adverse events, including mortality and major complications. This combined approach allowed more refined risk stratification and supported personalised treatment planning in TAVI care [21]. A pilot TAVI study showed that two simple pre-procedural indices—ASA class and Clinical Frailty Scale—outperformed EuroSCORE II (AUC ~0.80 vs. ~0.66) and also stratified length of stay and costs into clinically meaningful tiers (30). Meta-analyses confirm the pattern: pooled discrimination around AUC 0.78–0.79 for mortality, consistently above traditional scores [36,37,38,39,40]. Methodological caveats recur—heterogeneity, limited external validation, and sparse calibration reporting [37,38,41].

#### 3.3.2. Complication Prediction: Conduction, Pacemaker, Bleeding, Stroke/CVE

Pre-procedural predictors of conduction injury and pacemaker need have been modeled using clinical and imaging inputs with good performance; epicardial fat, annular geometry, and calcification consistently emerge as important features [24,27]. Dedicated studies confirm this, showing that AI-based models analyzing clinical and imaging data can effectively identify patients at higher risk of conduction abnormalities and permanent pacemaker implantation, outperforming conventional methods [27]. A recent clinical-only study found XGBoost best among classical ML for predicting persistent LBBB, while GPT-4 with reasoning prompts performed competitively, highlighting an emerging LLM role in structured clinical prediction [42,43]. In addition, Cheilas et al. demonstrated that even with clinical parameters alone, ML and LLMs could forecast new onset LBBB after TAVI, with XGBoost showing strong predictive ability and GPT-4 matching or exceeding conventional ML performance [44]. For rare but devastating events, a deep-learning autoencoder predicted 30-day cerebrovascular events with AUC ~0.79 in a large multicenter cohort despite low event rates, underscoring AI’s utility in rare-event settings [45]. Other studies extended these findings: DL-based CT angiography analysis uncovered subtle imaging features predictive of adverse events [46], and a Random Forest model incorporating clinical, biochemical, and imaging data identified femoral artery size, annular angle, and valve calcification as key drivers of early complications such as bleeding, kidney injury, and vascular problems [47]. Several narrative/position reviews reinforce both the promise and the barriers—opacity, bias, data privacy, and the need for prospective validation and clearer reporting [6,7,48,49,50,51].

**Table 1:** Summary of studies included in the review.

Author (Year)	Study Type	AI Method/Model	Data Source (CT/Echo/Clinical/Other)	Main Findings	Clinical Relevance/Impact
Wang et al. (2023) [2]	Multicenter retrospective imaging study	End-to-end deep learning (segmentation + landmarking)	CT (pre-TAVR aortic root, annulus, coronaries)	Automated pipeline achieved Acc 0.989, Sens 0.979, Spec 0.986 for anatomical risk factors; standardized outputs and faster analysis.	Reduces variability, accelerates pre-TAVR planning, and reliable risk-factor detection.
Skalidis et al. (2025) [3]	Narrative review	AI analytics + XR (VR/AR) visualization	ECG, Echo, CT; XR-assisted procedural planning	Synthesizes AI for sizing/simulation and XR for complex anatomy/MDT planning; highlights validation/standardization gaps.	Frames opportunities and barriers for clinical adoption; guides MDT workflows.
Windecker & Tomii (2025) [4]	Perspective/Commentary	CT strategy + AI-enabled planning principles	CT (lifetime valve care, redo-TAVR planning)	Discusses CT-based insights for redo-TAVR and lifetime management; need for standardized, reproducible imaging pathways.	Strategic guidance for longitudinal TAVR care planning.
Zhang et al. (2024) [5]	Review	Rule-based + ML approaches across pathway	ECG, digital auscultation, Echo, CT	AI augments screening and planning; bias/validation concerns emphasized.	Supports earlier detection and more consistent planning; underscores governance needs.
Watson et al. (2022) [6]	Review (cardiology AI)	Neural networks, computer vision	Imaging + EHR streams	Outlines gains in workflow efficiency and decision support; need for clinician–AI collaboration.	System-level rationale for adoption and training.
Henein et al. (2024) [7]	Focused review (TAVR)	Various supervised ML/DL	Pre/post-TAVR imaging + clinical data	AI predicts leaflet dysfunction, stroke, pacemaker, mortality; flags opacity/bias/privacy barriers.	Identifies promising applications with caveats for real-world use.
Zhang et al. (2022) [8]	Imaging reconstruction study	DL-based spiral CT 3D reconstruction	CT (pre-TAVI)	Improved SSIM/PSNR and ~90% correct valve sizing with high sensitivity/specificity.	Enhances image quality and valve sizing confidence.
Saitta et al. (2023) [9]	Retrospective imaging/technical validation	DL segmentation + landmark detection	CT (aortic root morphology)	Dice ~0.93; sub-millimeter landmark error (annulus/STJ); automated morphology extraction.	Reliable automated measurements for TAVI sizing/orientation.
Krüger et al. (2022) [10]	Technical pipeline/feasibility	Cascaded CNNs for segmentation and orientation	CT (aortic root analysis)	Runtime < 30 s; annulus diameter within ~2 mm of expert; robust orientation.	Near-real-time automated planning support.
Boeckling et al. (2025) [11]	Observational cohort (biomarkers)	Statistical and ML risk modeling	Serum ECM markers (incl. TIMP-1), hs-cTnT	TIMP-1 independently predicted 2-year mortality; combined with hs-cTnT outperformed STS-PROM.	Biomarker integration can refine TAVR risk stratification.
Bernhard et al. (2024) [12]	Observational imaging cohort	Feature extraction/ML on 4D-CT myocardium	4D Cardiac CT	CT-derived myocardial metrics predicted reverse remodeling and clinical outcomes post-TAVR.	Adds tissue-level markers to prognostication.
Boninsegna et al. (2024) [13]	Validation vs. radiologists	Automated CTA measurements (AI)	CTA (9 vessel landmarks)	Within ±2 mm accuracy vs. humans; <2 min AI vs. >5 min readers; ±1 mm discrepancies near aortic valve.	Speeds reporting with acceptable accuracy; gains in workflow.
Tremamunno et al. (2025) [14]	Retrospective observational	Supervised ML (feature-selected models)	Planning CT + clinical	AI improved prediction of MACE during/after TAVR-planning CT vs. conventional.	Earlier identification of higher-risk candidates during planning.
Sun et al. (2024) [15]	Narrative/Topical review	Various DL tools (e.g., TAVI-PREP, 4TAVR)	CTA ± other modalities	Automated extraction of 22+ measures in ~2 min (high correlation), strong annulus metrics; needs broader external validation.	Supports standardization and speed; flags generalizability gaps.
Cadour & Dacher (2024) [16]	Commentary	Photon-counting CT + AI decision support	CCTA	Argues AI + photon-counting CCTA could obviate invasive CA in TAVR work-up for many.	Potentially reduces invasive testing burden.
Brendel et al. (2024) [17]	Feasibility/cohort	Photon-counting CT with AI support	CCTA in TAVR work-up	Improved CAD evaluation quality in TAVR candidates; feasibility shown.	Strengthens non-invasive CAD assessment pathway.
Zhang et al. (2024) [18]	Prospective/observational imaging	DL image reconstruction (DLR)	CT (pre-TAVI)	Improved image quality and diagnostic performance with reduced contrast and dose.	Safer imaging (lower dose/contrast) without sacrificing accuracy.
Rouhollahi et al. (2023) [19]	Technical tool/software package	Deep learning segmentation and reconstruction (digital twins)	CT (aortic stenosis)	CardioVision generated patient-specific digital twins and calcification maps.	Enables simulation, planning, and device testing in silico.
Pekař et al. (2024) [20]	Observational cohort	DL body composition	CT (L3 SMA, fat density)	Low skeletal muscle index and higher fat density predicted poorer survival after TAVR.	Opportunistic prognostics from routine CT.
van Erck et al. (2024) [21]	Observational cohort	DL assessment of muscle quality	CT (procedural scan)	Low muscle quality predicted 1-year mortality in severe AS.	Frailty proxy from CT to refine risk.
Zsarnoczay et al. (2025) [22]	Retrospective cohort	AI-derived LACI (automated LA/LV volumes)	CTA (automated volumetry)	LACI ≥ 43.7% independently predicted mortality over ~2 years, including preserved EF.	Adds independent prognostic signal beyond STS-PROM.
Paukovitsch et al. (2025) [23]	Observational cohort	Automated vBMD estimation (AI)	Opportunistic CT (thoracic vertebrae)	Thoracic vBMD AUC ~0.96 for osteoporosis; osteoporosis is linked to worse 1-year survival.	Frailty/osteoporosis screening within routine TAVR CT planning.
Weferling et al. (2022) [24]	Observational cohort	Automated epicardial fat quantification	CT (pre-TAVI)	Higher epicardial fat is associated with baseline AV block and higher PPM rates.	Biologic risk layer for conduction injury.
Dasi et al. (2023) [25]	Modeling/computational	AI hemodynamic modeling	Clinical and hemodynamic features	Predicted post-TAVR pressure gradients consistent with physiologic patterns.	Assists device selection and expectation setting.
Busto et al. (2023) [26]	Algorithm validation (post-TAVR)	Automated 4DCT quantification (AI)	4DCT (prosthesis dynamics)	Automated prosthesis volume/area/displacement across cycle; aligned with valve size patterns.	Objective tracking for post-TAVR assessment.
Busto et al. (2025) [27]	Technical/observational	Automated 4DCT deformation analytics	4DCT throughout cardiac cycle	Quantified deformation: potential link to long-term durability monitoring.	Foundation for durability surveillance post-implant.
Kojima et al. (2024) [28]	Prospective imaging cohort	Deep-learning reconstruction (DLR) at low kV	CTA (low-tube-voltage)	Improved CNR, lower noise; preserved annulus measurement fidelity vs. HIR/MBIR.	Enables dose reduction without losing sizing accuracy.
Shao et al. (2025) [29]	Protocol/imaging cohort	Dual DLR combination (AI-IR)	CT on 8 cm detector scanner	≈50% dose reduction with equal/better quality for valve and access assessment.	Safer scanning for elderly/comorbid TAVR population.
Vaitkus et al. (2014) [30]	Protocol optimization	Low-kV strategy (not AI)	CCTA + aorto-iliac CTA	70–100 kV protocols preserved quality and enabled large dose savings; accurate annulus sizing.	Baseline for later AI-DLR dose reductions.
Li et al. (2025) [31]	Prospective cohort	AI Iterative Reconstruction (AI-IR)	Low-dose aortic CTA (access assessment)	AI-IR produced higher signal/contrast with least noise at lower dose.	Improves safety and confidence for access planning.
Mehier et al. (2024) [32]	Diagnostic cohort	DL classification/CAD exclusion	CCTA in TAVR work-up	Sensitivity 100% and NPV 100% for >50% stenosis; PPV ~39%.	If negative, it may obviate invasive angiography pre-TAVR.
Baeßler et al. (2023) [33]	Topical review	Multiple AI tools along pipeline	CCTA (CT-FFR, perfusion, risk scores)	AI assists acquisition→analysis; supports dose reduction and decision-making.	Consolidates best practices for CCTA-led work-up.
Agasthi et al. (2021) [34]	Retrospective cohort	Gradient Boosting Machine	Clinical and procedural variables	Outperformed TAVI-2/CoreValve scores for 1-year mortality prediction.	Sharper long-term risk identification.
Zisiopoulou et al. (2024) [35]	Pilot cohort	Supervised ML (with ASA, CFS)	Clinical pre-procedural data	AUC ~0.80 for 1-year mortality vs. EuroSCORE II ~0.66; stratified LOS and costs.	Simple clinical metrics enable accessible risk stratification.
Sazzad et al. (2024) [36]	Systematic review and meta-analysis	Aggregate across ML/DL approaches	Multi-study, multi-modality	Pooled AUC ~0.79 for mortality; exceeds conventional risk scores.	Evidence base for adopting ML prognostics.
Sulaiman et al. (2025) [37]	Systematic review	Multiple ML models	Clinical and imaging across studies	ML consistently superior for survival, pacemaker, MACE; urges external validation.	Synthesizes breadth of applications and gaps.
Zaka et al. (2025) [38]	Systematic review and meta-analysis	ML vs. traditional methods	Aggregated cohorts (~30k pts)	ML AUC ≈ 0.79 vs. ~0.68 for conventional scores (in-hospital/30d/1y mortality).	Confirms superiority across time horizons.
Lachmann et al. (2022) [39]	Methodologic cohort	Pre-trained CNN (VGG-16) features; unsupervised clustering	Doppler outflow velocity profiles (AS)	Unsupervised separation of flow phenotypes from VGG-16 features.	Supports AI-aided phenotyping beyond imaging morphology.
Toggweiler et al. (2024) [40]	Software feasibility	Fully automated AI planning software	CTA (multi-center)	End-to-end planning outputs; feasibility in clinical workflows.	Streamlines planning and standardizes measurements.
Shojaei et al. (2025) [41]	Systematic review and meta-analysis	Multiple ML/DL	43 studies; >360k TAVR pts	Mortality AUC ~0.78; conduction AUC ~0.75; imaging/biomarkers improve models.	Comprehensive evidence of AI’s prognostic value.
Yannakula et al. (2025) [42]	Narrative review	Real-time intra-procedural AI guidance	IVUS/OCT/fluoro/echo (concepts)	Landmark detection, device positioning concepts; integration/regulatory hurdles.	Future path for live guidance.
Vasileios et al. (2025) [44]	Clinical cohort (no prior LBBB)	ML classifiers (XGBoost, etc.) and LLMs	Clinical pre-implant variables	Persistent LBBB ~15%; XGBoost best; GPT-4 competitive via reasoning prompts.	Risk-flagging to tailor device choice/implant depth/monitoring.
Okuno et al. (2021) [45]	Multicenter cohort	DL autoencoder (rare-event prediction)	Clinical and imaging (TAVR)	AUC ~0.79 for 30-day cerebrovascular events despite low incidence.	Enables CVE risk flagging and preventive strategies.
Zheng et al. (2021) [46]	CTA DL study	DL feature extraction/classification	CTA (pre-TAVI and complications)	Identified CT image markers predictive of complications beyond standard review.	Sharper risk stratification from CTA.
Kurmanaliyev et al. (2025) [47]	Cohort (n = 224)	Random Forest and class imbalance handling; SHAP	Clinical, imaging, and labs	Early 30-day complications predicted; key features: femoral diameter, annulus angle, calcification.	Pre-procedural triage for safety planning.
Bamford et al. (2024) [48]	State-of-the-art review	Multiple ML/DL modalities	Screening→planning→procedure	AI stethoscopes, ECG rule-out tools; automated TAVI sizing/placement; early robotics.	Broad overview of maturing use-cases in valve disease.
Kwiecinski et al. (2023) [49]	Multicenter registry	Supervised ML (various)	Clinical ± imaging	Improved 1-year mortality discrimination vs. conventional scores.	Supports ML adoption for routine outcomes.
Jacquemyn et al. (2025) [50]	Systematic review and meta-analysis	ML prognostics (multiple methods)	Aggregated literature	Strong performance, but reproducibility/reporting limitations impede translation.	Roadmap for better reporting/validation.
Herrero-Brocal et al. (2025) [51]	Program evaluation	AI-supported telemonitoring model (TeleTAVI)	Remote monitoring data	Enabled early discharge with close follow-up; feasibility and safety were described.	Resource optimization and patient monitoring.
Scuoppo et al. (2025) [52]	Modeling/in silico	Statistical shape modeling and ML	CT (aortic root with calcification)	Generated virtual TAVI cohort resembling real anatomies; accurate sizing and gradient estimation.	Accelerates testing and planning with synthetic anatomies.

#### 3.3.3. Multimodal and Biomarker-Integrated Risk

Integrating imaging, clinical, and circulating biomarkers can further boost prediction. Machine-learning models combining TIMP-1 and high-sensitivity troponin T outperformed STS-PROM for 2-year mortality, suggesting extracellular-matrix remodeling and myocardial injury markers add orthogonal signal [11]. Similarly, CT-derived phenotypes (sarcopenia, fat quality, LACI, osteoporosis) complement clinical scores and may refine selection, counseling, and follow-up planning [11,22,23].

### 3.4. Emerging Directions: XR, Digital Twins, Virtual Cohorts, and Real-Time Guidance

#### 3.4.1. XR-Assisted Planning and Team Decision-Making

XR virtual reality (VR)/augmented reality (AR) is being explored for complex anatomy review, simulation of valve placement, and enhancing MDT communication. Current evidence emphasizes feasibility and perceived decision support, with adoption limited by validation, standardization, and workflow integration hurdles [2,3].

#### 3.4.2. Virtual Cohorts and In Silico Evaluation

Statistical shape models and synthetic cohorts replicate the breadth of aortic-root morphologies (and calcification patterns), enabling rapid “what-if” testing of device sizing and procedural strategies. Early studies show high sizing accuracy and credible physiologic estimates (e.g., gradients), pointing toward scalable, lower-risk evaluation pathways that complement clinical trials [19,52].

#### 3.4.3. Toward Intra-Procedural AI

Narrative syntheses suggest real-time AI for landmarking, complication detection, and deployment optimization across interventional cardiology, including structural heart procedures. Translation will depend on fast, reliable processing, user-centric UI, and prospective evaluation demonstrating safety and benefit [42].

AI 51 articles regarding studies on application of AI models on TAVI procedures, the role of artificial intelligence in the new digital era of Cardiology for all the stages of the procedure is highlighted (all applications as described in the text before are summarized on Table 2). Specifically, applications for decision making, risk stratification, outcomes prediction, imaging modalities optimization, and procedural planning were reported in the literature. What is notable is the fact that AI models were employed to integrate a wide range of features, such as baseline clinical characteristics, imaging data, laboratory testing and biomarkers, and procedural factors. This capacity to integrate numerous different features into a single model represents a key element of AI over traditional risk scores, which have limitations due to static variables. AI models can capture complex relationships and common patterns between many factors, offering a more holistic risk stratification.

A comparative analysis between AI-based prediction models and established clinical risk scores such as EuroSCORE II and STS-PROM highlights the superior capability of AI in capturing complex, nonlinear relationships among multiple clinical variables. As noted by Cersosimo et al., AI models can process large, multidimensional datasets, providing greater predictive accuracy than conventional scores. However, their role should remain that of a clinical decision-support tool that complements rather than replaces physician expertise, in accordance with the latest EAPCI and ESC AI Task Force recommendations [53].

Furthermore, a study investigated the prognostic value of exercise stress echocardiography with tissue Doppler imaging in 90 patients with mild-to-moderate asymptomatic aortic stenosis beyond its routine indication for evaluation of low-flow-low gradient aortic valve stenosis. It demonstrated that patients exhibiting a stress-induced increase in E/e′ ratio ≥ 15 had significantly higher rates of adverse cardiac events, including heart failure hospitalization and cardiovascular death, during follow-up. The findings suggest that diastolic dysfunction unmasked during stress is a powerful predictor of poor outcomes, emphasizing the importance of dynamic assessment in risk stratification beyond resting parameters in aortic stenosis [43]. Considering this example, AI algorithms could indeed play a valuable role in evaluating aortic stenosis (AS) patients undergoing exercise stress echocardiography (ESE) before and after TAVR. By integrating imaging parameters, hemodynamic data, and clinical variables, AI can automate the quantification of stress-induced changes, such as E/e′ ratio, longitudinal strain, and valve gradients with higher accuracy and reproducibility. Machine-learning models could also identify subtle patterns of diastolic dysfunction or ventricular–arterial coupling not evident through conventional analysis, enhancing pre-procedural risk stratification and post-TAVR functional assessment. In the future, explainable AI and multimodal data integration may allow personalized prediction of functional recovery and long-term outcomes following TAVR in AS patients.

AI has significantly enhanced the efficiency of TAVI procedures by automating complex imaging tasks, reducing analysis time, and standardizing measurements that traditionally required extensive manual input. Through advanced machine-learning and deep-learning models, patient selection has become more accurate, integrating clinical, imaging, and biomarker data to identify optimal candidates and anticipate procedural risks more effectively than conventional scores like EuroSCORE II or STS-PROM. Postoperatively, AI-driven predictive tools outperform traditional models in forecasting complications such as pacemaker need, stroke, and mortality, enabling personalized follow-up strategies and overall improvement in clinical outcomes and resource utilization.

Future studies are needed to assess the performance and efficacy of AI models in predicting the evaluated outcomes across the spectrum of cardiac procedures. Moreover, continued research is essential to determine the specific model that is more efficient and safer in each clinical context. Utilization of the AI tools could be applied to all types of clinical outcomes, such as hospital readmission rate, financial parameters, quality of life, and frailty. In addition, trials with larger populations are required to validate the AI models in real-world settings, assessing their impact on the decision-making process, and determining which tool is more reliable for each clinical scenario. Finally, taking into consideration that AI offers promising advantages in cardiovascular system disease and care, some notable challenges, such as incomplete medical databases, cost of computer networks, and the need for a standardized tool to validate the effectiveness and safety of these algorithms, is necessary to be addressed. Given the fact that cardiovascular procedures become increasingly complicated, the application of AI seems inevitable in order to maintain high-quality healthcare procedures, but also the role of human control on these features is crucial and should always be highlighted. The final decision for a more patient-centered medicine should be taken by doctors, after careful evaluation and assistance from AI algorithms, which can enhance all the parameters of the procedure, without replacing humans, but by becoming an assisting tool of utmost importance in every aspect of the Heart Team.

Future directions for AI in TAVI focus on improving transparency, collaboration, and clinical integration. Explainable AI (XAI) aims to make algorithmic decisions more interpretable, allowing clinicians to understand how predictions are generated and to maintain clinical accountability. Federated AI offers a way to train models across multiple institutions without sharing sensitive patient data, enhancing generalizability while preserving privacy under GDPR standards. Finally, seamless integration into clinical workflows through real-time decision-support tools, automated imaging pipelines, and interoperability with electronic health records will be essential to ensure that AI systems complement clinician expertise and improve procedural safety, efficiency, and patient outcomes.

## 4. Limitations

Despite its potential, AI implementation in TAVI faces several challenges. Many models are trained on limited or single-center datasets, reducing their generalizability and clinical reliability. The lack of transparency in deep-learning algorithms also limits physician trust and interpretability, minimizing human factors. Data heterogeneity, incomplete registries, and variations in imaging protocols further restrict consistency across centers. Moreover, compliance with GDPR and the forthcoming EU AI Act imposes strict requirements for data protection, accountability, and human oversight. Finally, the high cost of infrastructure, need for specialized expertise, and absence of standardized validation frameworks continue to slow large-scale clinical adoption.

## 5. Conclusions

In conclusion, the results of our review emphasize the role of artificial intelligence in TAVI procedures, with many applications on imaging modalities, procedure planning, decision making, and risk stratification for outcomes. These models could be utilized by physicians for optimization of the procedure and prediction of complications in high-risk patients. AI algorithms could revolutionize the Heart Team decision making process, being not only a tool for patient evaluation, but an active member of the Team with applications to analyze and optimize all stages of the TAVI procedure, guide decision making and predict outcomes, and, with the contribution and evaluation of information from all human members of the team, enhance even more the patient-mediated medicine/interventions. Moreover, it is essential to highlight the need for proper ethical regulations to ensure safe AI integration in cardiology. AI interpretability ensures transparency, while data protection under GDPR protects patient privacy. The forthcoming EU AI Act introduces a risk-based approach emphasizing safety, accountability, and human oversight, ensuring AI tools remain transparent, secure, and clinically trustworthy.

## Figures and Tables

**Table 2:** Summary of AI algorithms and their applications in TAVI Studies.

Algorithm Type	Representative Models/Methods	Main Application	Data Type Used	Performance Highlights/Key Findings
Deep Learning (DL)	CNNs, U-Net, VGG-16, Autoencoders	Automated CT segmentation, 3D/4D reconstruction, landmark detection	CT/4D-CT	Accuracy up to 0.98; sub-mm precision; reduced analysis time (<30 s); improved valve accuracy (~90%)
Machine Learning (ML)	Random Forest, Gradient Boosting XGBoost, SVM	Prediction of mortality, conduction abnormalities complications (stroke, pacemaker, bleeding)	Clinical +Imaging+ Biomarker data	AUC 0.78–0.90; superior to EuroSCORE II/STS-PROM in short-and long term prediction
Hybrid/Multimodal Models	Combined ML+ imaging/ biomarker integration	Risk stratification and outcome prediction using clinical+ CT+ biomarker data	Clinical+ Imaging+ Biomarkers data	TIMP-1 + hs-cTnT model outperformed STS-PROM for 2-year mortality enhanced prognostic power
Explainable ML models	SHAP-enhanced Random Forest, feature selection	Identification of drivers of early complication (bleeding, vascular injury)	Clinical+ Imaging	Highlighted key anatomical predictors (femoral diameter, annular angle, calcification)
Digital Twin & Simulation Models	CardioVision, Statistical Shape Modeling	Patient-specific 3D reconstruction and procedural simulation	CT Angiography	40–50% dose reduction with preserved or improved diagnostic accuracy
LLMs/NLP Models	GPT-4, reasoning-prompted models	Structural clinical prediction, post-TAVI conduction outcomes	Clinical Variables	Comparable or superior to classical ML for LBBB prediction; supports reasoning-based risk forecasting
XR/Augmented Reality Integration	AI-assisted VR/AR visualization	Procedural planning and Heart Team simulation	CT + XR data	Enhanced MDT communication and complex anatomy assessment; requires further validation
AI Reconstruction Algorithms	Deep Learning Reconstruction (DLR), AI Iterative Reconstruction (AI-IR)	Image quality enhancement, radiation dose reduction	CT Angiography	40-50% dose reduction with preserved or improved diagnostic accuracy

## Data Availability

All Data used in our study are widely available in the two platforms that were used for conduction of our review: Pubmed and Google Scholar (PubMed, Scholar Google).
